# Two Pathways Recruit Telomerase to *Saccharomyces cerevisiae* Telomeres

**DOI:** 10.1371/journal.pgen.1000236

**Published:** 2008-10-24

**Authors:** Angela Chan, Jean-Baptiste Boulé, Virginia A. Zakian

**Affiliations:** Department of Molecular Biology, Princeton University, Princeton, New Jersey, United States of America; Fred Hutchinson Cancer Research Center, United States of America

## Abstract

The catalytic subunit of yeast telomerase, Est2p, is a telomere associated throughout most of the cell cycle, while the Est1p subunit binds only in late S/G2 phase, the time of telomerase action. Est2p binding in G1/early S phase requires a specific interaction between telomerase RNA (TLC1) and Ku80p. Here, we show that in four telomerase-deficient strains (*cdc13-2*, *est1Ä*, *tlc1-SD*, and *tlc1-BD*), Est2p telomere binding was normal in G1/early S phase but reduced to about 40–50% of wild type levels in late S/G2 phase. Est1p telomere association was low in all four strains. Wild type levels of Est2p telomere binding in late S/G2 phase was Est1p-dependent and required that Est1p be both telomere-bound and associated with a stem-bulge region in TLC1 RNA. In three telomerase-deficient strains in which Est1p is not Est2p-associated (*tlc1-SD*, *tlc1-BD*, and *est2Ä*), Est1p was present at normal levels but its telomere binding was very low. When the G1/early S phase and the late S/G2 phase telomerase recruitment pathways were both disrupted, neither Est2p nor Est1p was telomere-associated. We conclude that reduced levels of Est2p and low Est1p telomere binding in late S/G2 phase correlated with an *est* phenotype, while a WT level of Est2p binding in G1 was not sufficient to maintain telomeres. In addition, even though Cdc13p and Est1p interact by two hybrid, biochemical and genetic criteria, this interaction did not occur unless Est1p was Est2p-associated, suggesting that Est1p comes to the telomere only as part of the holoenzyme. Finally, the G1 and late S/G2 phase pathways for telomerase recruitment are distinct and are likely the only ones that bring telomerase to telomeres in wild-type cells.

## Author Summary

Duplication of linear DNA is complicated by the fact that conventional DNA polymerases cannot copy their ends. From yeasts to humans, replication of DNA ends, called telomeres, is accomplished by a telomere-dedicated reverse transcriptase called telomerase that uses its RNA subunit as a template. We show that there are two genetically distinct pathways that recruit yeast telomerase, Est2p, to telomeres in a cell cycle–dependent manner. Only one of the two pathways, the pathway operating late in the cell cycle, is required for telomere maintenance. In telomerase-deficient strains, the amount of Est2p that is telomere-bound late in the cell cycle is about 50% of wild type levels. Maintenance of functional levels of Est2p late in the cell cycle requires that Est1p, another telomerase subunit, be telomere-bound. In addition, Est1p must be associated with Est2p via an interaction between it and telomerase RNA. Human telomerase is not active in most somatic cells, but is critical for stem cell longevity. Even a modest reduction in telomerase has a serious impact on human health. The sensitivity of yeast to reduced levels of telomere-associated telomerase may help us understand why human stem cells require high levels of telomerase for their survival.

## Introduction

Telomerase is a specialized reverse transcriptase that lengthens the 3′ end of telomeric DNA. In *Saccharomyces cerevisiae*, the template for telomere elongation is a short stretch within the 1158 base TLC1 telomerase RNA. Est2p is the *S. cerevisiae* telomerase catalytic subunit, while Est1p and Est3p are two telomerase subunits whose roles in telomerase action are less well understood. Although Est2p and TLC1 RNA are sufficient for telomerase catalytic activity *in vitro*, all three *EST* proteins, as well as TLC1 RNA are required *in vivo*. Telomerase deficient strains such as *tlc1Δ*, *est2Δ*, *est1Δ*, and *est3Δ* are viable but slowly lose telomeric DNA (reviewed in [1]). After 50–100 generations, when telomeres are very short, chromosome loss increases in these strains, and most cells in the population die, a collection of behaviors known as the *e*ver *s*horter *t*elomere (*est*) phenotype [2].

The key events in *S. cerevisiae* telomere replication and processing occur in late S/G2 phase. Most of the ∼300 bp yeast telomere is replicated by semi-conservative DNA replication, which occurs very late in S phase. After semi-conservative replication, C-strand resection generates ∼50–100 base G-tails at both ends of DNA molecules. These G-tails are repaired by C-strand resynthesis prior to mitosis [3]–[5]. Telomerase lengthening of telomeres also occurs late in the cell cycle [6],[7].

Cdc13p is a single-strand TG_1–3_ sequence specific DNA binding protein [8],[9] that associates *in vivo* with the G-tails that constitute the very ends of yeast chromosomes [10],[11]. Although the Cdc13p complex has an essential role in protecting telomeres from degradation [12]–[14], there are also alleles of *CDC13*, such as *cdc13-2*, that have normal end protection activity but confer an *est* phenotype [9]. Cdc13p and Est1p interact by two-hybrid, co-immuno-precipitation [15], and genetic criteria [16]. Moreover, fusions between the DNA binding domain of Cdc13p and Est2p can maintain telomeres in the absence of Est1p [17]. Together these data suggest that Est1p acts by recruiting Est2p to the telomere in late S/G2 phase. This recruitment is thought to occur via a specific interaction between Est1p and Cdc13p that is lost in telomerase defective *cdc13-2* cells. The association of Est3p with Est2p is Est1p-dependent [18].

In previous work, we used chromatin immuno-precipitation (ChIP) to test different aspects of this recruitment model [19]. Consistent with the model, Est1p binding to telomeres is limited to late S/G2 phase, and Cdc13p binding, which occurs throughout the cell cycle, increases enormously at this time, concomitant with the appearance of long G-tails. However, Est2p is telomere associated throughout most of the cell cycle, not just in late S/G2 phase as the model predicts. The high Est2p binding in late S/G2 phase is reduced by ∼50% in the telomerase deficient *cdc13-2* strain, while Est2p binding earlier in the cell cycle is unaffected. The telomere association of Est2p at times when telomerase is not active is also inferred by fluorescent in situ hybridization, which shows co-localization of TLC1 telomerase RNA with telomeres in G1 and S phase cells [20]. Unexpectedly, Est1p binding was equivalent in wild type (WT) and *cdc13-2* cells, although the signal to noise ratio for Est1p in these early ChIP experiments was low [19].

A more sensitive ChIP assay was used to determine the requirements for Est2p binding in G1 and early S phase [21]. Mutations in *TLC1* (*tlc1Δ48*) or *YKU80* (*yku80-135i*) that disrupt the ability of TLC1 RNA to interact with Ku80p both *in vitro* [22],[23] and *in vivo* [21] eliminate Est2p telomere binding in G1 and early S phase [21]. In addition, Est2p and Est1p telomere binding in late S/G2 phase is reduced to ∼50% of WT levels in these mutants. Although telomeres in *tlc1Δ48* and *yku80-135i* cells are shorter than in WT cells, neither strain has an *est* phenotype [22],[23]. Thus, telomerase binding to yeast telomeres in G1 and early S phase is not required for telomere maintenance.

Here we extend the analysis of the requirements for Est2p telomere binding. Several additional *est* mutations, *est1Δ*, *tlc1-SD*, and *tlc1-BD*, had the same Est2p telomere binding profile as *cdc13-2* cells. Thus, reduced levels of Est2p binding specifically in late S/G2 phase correlated with an *est* phenotype, while a WT level of Est2p binding in G1 was not sufficient to maintain telomeres. The specific interaction between TLC1 and Est1p that is lost in *tlc1-SD* and *tlc1-BD* cells [24], resulted in low, but detectable, Est1p telomere binding as did the *cdc13-2* mutant. Est1p telomere binding was not detected in an *est2Δ* strain. Together, these data show that WT levels of Est2p telomere binding in late S/G2 phase require that Est1p bind telomeres, a binding that requires a specific interaction between Est1p and TLC1 RNA and that is reduced in *cdc13-2* cells. No Est2p or Est1p was detected at telomeres when both the G1/early S and the late S/G2 phase pathways for Est2p recruitment were disrupted (as in *tlc1Δ48 cdc13-2* or *yku80-135i tlc1-SD* double mutant cells). Thus, these pathways are likely the only ones that recruit telomerase to yeast telomeres *in vivo*.

## Results

### Est2p Telomere Binding in Late S/G2 Phase (but not G1 Phase) Is Reduced in *est1Δ* Cells

Chromatin immuno-precipitation (ChIP) was used to determine the telomere association of proteins involved in telomere maintenance in WT and mutant cells. We used strains in which the protein being studied was multiply epitope tagged at its endogenous locus and was the only form of the protein in the cell. Functionality of epitope tagged proteins was determined by their effects on telomere length and other telomere phenotypes. In earlier experiments, we used a Myc-tagged Est2p that was not fully functional as telomeres were ∼50 bps shorter in its presence [19]. When this Myc-tagged Est2p was introduced into an *est1*Δ strain, cells senesced so rapidly that it was not possible to determine whether Est2p binds telomeres in *est1Δ* cells.

The function of epitope tagged Est2p was improved by inserting a flexible linker of eight glycine residues between the carboxyl terminus of Est2p and multiple Myc epitopes (hereafter called Est2-G8-Myc) [21],[25]. This allele supports WT telomere length, does not senesce when combined with deletion of *YKU*, and *est1Δ* cells carrying this Est2-G8-Myc can be grown for 50 to 100 cell divisions before they senesce. This Est2-G8-Myc allele was used to determine if Est2p telomere binding is Est1p-dependent.

**Figure 1:**
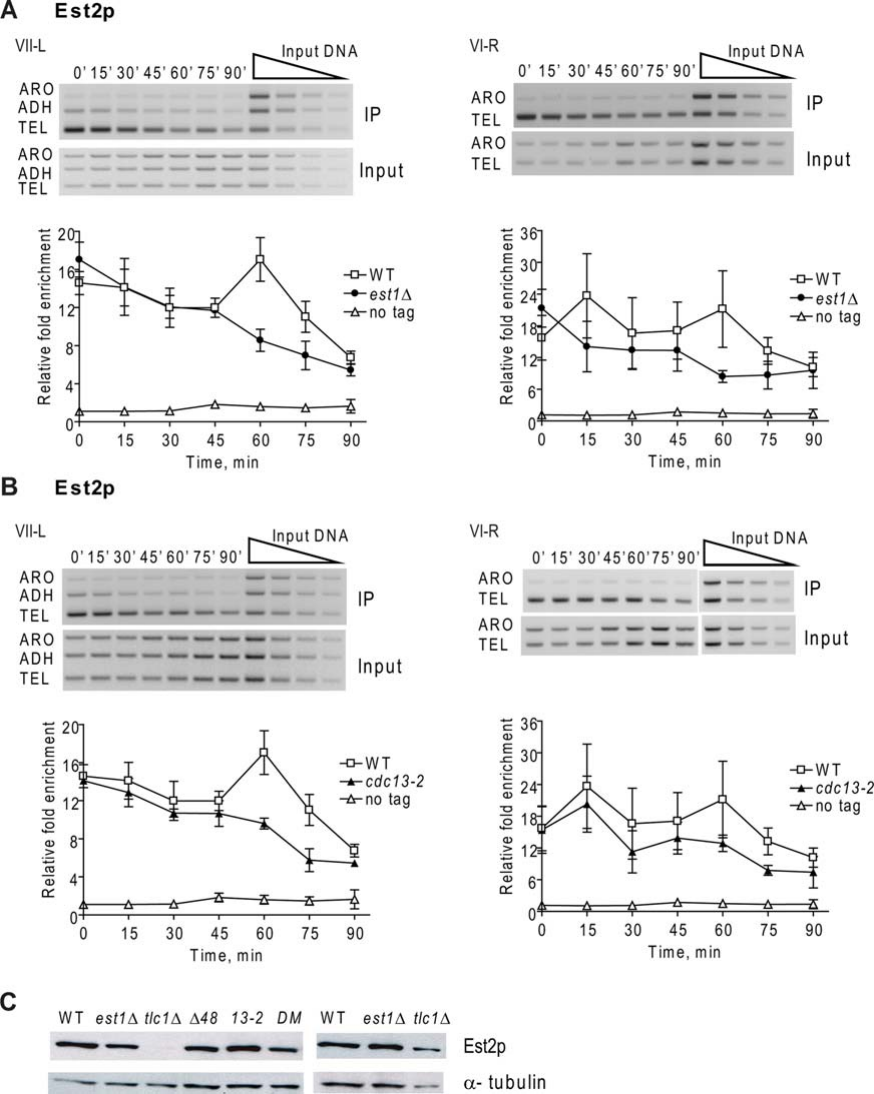
Est2p telomere binding in late S/G2 phase is reduced in *est1Δ* and *cdc13-2* cells. Cells expressing Est2-G8-Myc or lacking a Myc-tagged protein (no tag) were arrested in G1 phase. After release from the arrest, cells were grown at 24°C and samples taken at 15 min intervals for FACS and chromatin immuno-precipitation (ChIP). After DNA purification, PCR amplification was carried out with telomeric (TEL), subtelomeric (ADH), and non-telomeric (ARO) primers on DNA from immuno-precipitates (IP) or whole cell lysates (Input). Two-fold serial dilutions of input DNA established the linear range of the reactions (Input, top right). In this and subsequent figures, the agarose gels are representative data from mutant strains. For each time point, binding is expressed as the relative fold enrichment of TEL over ARO signal after normalization to input DNA. Error bars are ±1 standard deviations from ≥3 independent synchronies. A. Est2p binding to VII-L (left) and VI-R (right) telomeres in synchronous *est1Δ* (black circles) versus WT (white squares), or untagged (white triangles) cells. The values for Est2p binding to the VII-L telomere were not significantly different in WT versus *est1Δ* cells (P values >0.05), except at 60 (P value of 0.002), 75 (P = 0.02) and 90 (P = 0.04) minutes. Est2p binding to the VI-R telomere in WT versus *est1Δ* cells was significantly different only at 60 (P = 0.001) and 75 (P = 0.01) min. B. Est2p binding to VII-L (left) and VI-R (right) telomeres in synchronous *cdc13-2* (black triangles) versus WT (white squares), or untagged (white triangles) cells. Est2p binding to the VII-L telomere were significantly different in WT versus *cdc13-2* cells only at 60 (P = 0.01) and 75 (P = 0.02) minutes. At VI-R, binding was significantly different only at 75 minutes (P = 0.005) C. Western analyses of Est2p-G8-Myc or α-tubulin levels in extracts from WT and mutant strains with duplicate extracts prepared from independent colonies. The lanes labeled *Δ48* are from *tlc1Δ48* cells; *13-2* is *cdc13-2*; DM is double mutant *tlc1Δ48 cdc13-2*. Est2p-G8-Myc in lane 3 (*tlc1Δ*) was detectable upon longer exposure; see last lane that has protein sample from another *tlc1Δ* isolate and Figure 4C.

Otherwise isogenic WT or *est1Δ* cells expressing Est2-G8-Myc were arrested in late G1 phase with alpha factor (0 minute time point) and then released into the cell cycle at 24°C. Samples were taken at 15 min time intervals and processed for FACS to determine position in the cell cycle, and by ChIP to determine Est2p association with telomeric DNA. Under these conditions, cells were in G1 phase at 0 and 15 min, in S phase at 30, 45, and 60 minutes, and in G2/mitosis at 75 and 90 min [19],[21] (and data not shown). None of the mutations or epitope tagged proteins had reproducible effects on cell cycle progression [19],[21] (and data not shown). ChIP samples were analyzed by quantitative multiplex PCR using primer pairs specific for the modified VII-L telomere (TEL), sub-telomeric VII-L DNA (ADH), or a sequence far from a telomere (ARO) (Figure 1A, B, left). Alternatively we examined association with the native VI-R telomere and ARO (Figure 1A, B right). For all synchronies, a representative gel from one of the three or more independent synchronies is shown. Fold enrichment is binding at telomere VII-L or VI-R relative to binding at ARO and normalized to input DNA [21]. Graphs are the compiled data from the three or more independent synchronies for a given strain; error bars are one standard deviation from the average for each time point.

As shown previously [19],[21], in WT cells Est2p had high telomere association in G1 and early S phase (0 through 30 min), a modest decline in mid-S phase, a second peak in late S/G2 phase (60 minutes), and then a decline in association as cells progressed to the end of the cell cycle (Figure 1A, white squares). Est2-G8-Myc association with the VII-L telomere in *est1Δ* cells was similar to what was seen in WT cells except that binding in late S/G2 phase (60–90 min) was significantly reduced (Figure 1A left, black circles; see figure legends for P values). A similar pattern of Est2-G8-Myc binding in the absence of Est1p was seen at telomere VI-R (Figure 1A right). This pattern of binding was very similar to that seen for Est2-G8-Myc binding in another telomerase defective strain, *cdc13-2* (Figure 1B, black triangles). The results for *cdc13-2* presented here with Est2-G8-Myc are similar to our published data carried out with the less functional Est2p-Myc and using different quantitation methods [19]. Reduced Est2p binding in *est1Δ* and *cdc13-2* cells was not due to reduced abundance of Est2p (Figure 1C, lanes labeled *est1Δ* and *13-2*). We conclude that Est1p is not essential for Est2p telomere binding but is required for WT levels of Est2p binding in late S/G2 phase.

### Est1p Telomere Binding Is Low in *cdc13-2* Cells

In addition to changing our tagging strategy, a series of technical changes were made in the ChIP protocol that increased the signal to noise ratio in these experiments [21]. These changes were particularly important for Est1p since in our earlier work, the association of Est1p with telomeres in late S phase was enriched only five fold over background [19]. Under the conditions of these previous experiments, Est1p bound equally well to telomeres in WT and *cdc13-2* cells. Since the result was surprising (see introduction), we redid this experiment using our more sensitive ChIP methods.

**Figure 2:**
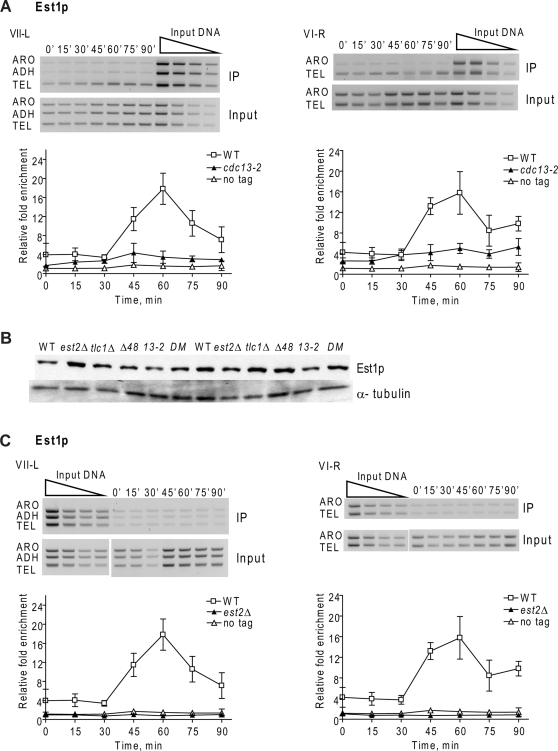
Est1p telomere binding is greatly reduced in synchronous *cdc13-2* cells and eliminated in *est2Δ* cells. Methods and symbols are as described in the Figure 1 legend except that cells expressed Est1-Myc. A. Est1p binding to VII-L (left) and VI-R (right) telomeres in synchronous *cdc13-2* (black triangles) versus WT (white squares), or untagged (white triangles) cells. Est1p binding to the VII-L telomere was significantly higher in *cdc13-2* cells than in the no-tag control at times of peak Est1p binding (P values ranged from 0.0003 at 60 min to 0.035 at 90 min). Est1p binding to the VI-R telomere was significantly different from the no-tag control at all time points (P values ranged from 0.0006 at 60 min to 0.0065 at 90 min.). B. Western analyses of Est1p-Myc versus α-tubulin levels in extracts from WT and mutant strains. The lanes labeled *Δ48* are from different *tlc1Δ48* colonies; *13-2* is *cdc13-2*; DM is double mutant *tlc1Δ48 cdc13-2*. C. Est1p binding to VII-L (left) and VI-R (right) telomeres in synchronous *est2Δ* (black triangles) versus WT (white squares), or untagged (white triangles) cells. Est1p binding to the VII-L and VI-R telomeres in *est2Δ* cells was modestly higher than the no-tag control only at 45 (P = 0.04 VII-L; 0.013, VI-R) and 60 min (P = 0.04, VII-L; 0.03, VI-R).

As shown previously [19], in WT cells, Est1p binding to the VII-L telomere peaked in late S phase (60 min) (Figure 2A, left, white boxes). With the improved ChIP protocols, peak Est1p binding was 20-fold above background. Contrary to our previous results, Est1p telomere binding to the VII-L telomere in *cdc13-2* cells was much lower than WT, only ∼4 fold above background (Figure 2A, left, black triangles). Although this binding was low, it was significantly higher than the signal with the no-tag control strain at all time points (Figure 2A). Reduced but significant Est1p binding was also seen at telomeres VI-R (Figure 2A, right) and XV-L (data not shown) in *cdc13-2* cells. Western analysis demonstrated that this reduced Est1p telomere binding was not due to an effect of the *cdc13-2* mutation on Est1p levels (Figure 2B, compare WT lanes to lanes labeled 13-2). We attribute the difference in these results compared to our earlier studies to the increased sensitivity of the current ChIP assay.

### Est1p Telomere Binding Is Est2p-Dependent

By multiple criteria, Est1p interacts with Cdc13p, and this interaction is thought to recruit Est2p to telomeres (see introduction). If Est1p binding depends solely on its ability to interact with Cdc13p, Est1p might bind telomeres even in an *est2Δ* strain. However, using synchronous cells, Est1-Myc binding to telomeres VII-L and VI-R in *est2Δ* cells (Figure 2C, black triangles) was very low, similar to background levels (Figure 2C, no tag, white triangles). The very low Est1p at *est2Δ* telomeres supports the interpretation that the signal for Est1p telomere binding in *cdc13-2* cells (Figure 2A) was real. The absence of Est1p binding was not due to reduced levels of Est1p in *est2Δ* cells (Figure 2B, compare WT to *est2Δ* lanes).

### Est1p Telomere Binding Is Low in the Absence of a Specific Interaction between Est1p and TLC1 RNA

TLC1 RNA is immuno-precipitated with Est1p [26],[27]. RNA structure analysis identified a potential stem-bulge region contained within nucleotides 600 to 669 that is conserved among different yeasts [24]. Both the putative 9 bp stem and the 5 nucleotide bulge are essential for telomerase function *in vivo*. *TLC1* alleles that delete either the bulge (*tlc1*-*BD*, bulge deletion allele) or that reduce base-pairing in the stem (*tlc1-SD*, stem disruption allele) yield an *est* phenotype. The stem-disruption compensatory mutation (*tlc1-SC*, stem compensatory), which restores the potential for stem formation in the *tlc1-SD* allele, maintains WT length telomeres. Several lines of evidence indicate that the stem-bulge region interacts with Est1p. Est1p (but not Est2p) over-expression suppresses the *est* phenotype of *tlc1-BD* cells. Moreover, neither TLC1-BD RNA nor TLC1-SD RNA immuno-precipitates with Est1p. The loss of interaction with TLC1 RNA is specific for Est1p as both mutant RNAs immuno-precipitate with Est2p [24].

**Figure 3:**
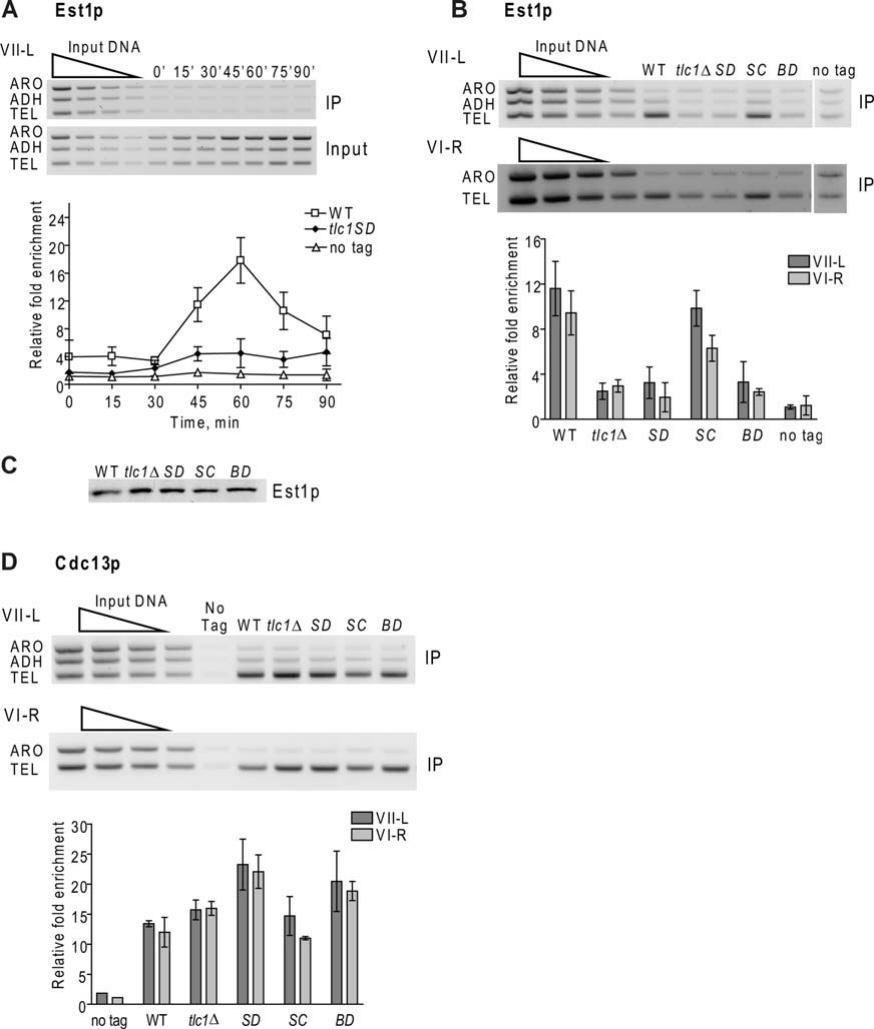
Est1p telomere binding is low in the absence of the stem-bulge region of TLC1 RNA. Methods and symbols are as described in legend of Figure 1 except that cells expressed Est1-Myc and for panels B–D, asynchronous log phase cells were analyzed. A. Est1p binding to VII-L telomere in synchronous *tlc1-SD* (black diamonds) versus WT (white squares), or untagged (white triangles) cells. Although Est1p binding from 30 through 90 min was higher in *tlc1-SD* cells than in the no tag control, the difference was significant (P = 0.045) only at 45 min. B. Est1p binding to VII-L (top) or VI-R (bottom) telomeres in WT and mutant asynchronous cells. Bar graphs show average Est1p association with telomere VII-L (dark grey) or VI-R (light grey) with error bars indicating ±one standard deviation from that average; abbreviations for strains are SD, *tlc1-SD*; SC, *tlc1-SC*; BD, *tlc1-BD*. The level of binding in *tlc1-SC* cells was not significantly different from WT (P = 0.16, VII-L; 0.07, VI-R). The level of binding in both *tlc1-SD* and *tlc1-BD* cells was not significantly different from *tlc1Δ* cells (P values ranged from 0.22 to 0.29). The level of Est1p binding in *tlc1Δ* cells was significantly higher than in the no-tag control (P = 0.011, VII-L; 0.029, VI-R). Likewise, the level of Est1p binding in *tlc1-SD* (P = 0.03, VII-L; 0.13, VI-R) and *tlc1-BD* (P = 0.074, VII-L; 0.07, VI-R) was mostly significantly higher than the no-tag control. C. Western analyses of Est1p-Myc in extracts from WT and mutant strains. Abbreviations for strains are same as in panel B. D. Cdc13p binding to VII-L (top) and VI-R (bottom) telomeres in asynchronous mutant and WT cells. Bar graphs and symbols are as in panel B. Cdc13p binding was not significantly different in *tlc1-SD* versus *tlc1-BD* cells (P = 0.5, VII-L; 0.2, VI-R). Cdc13p binding was significantly higher than in WT at both telomeres in *tlc1-SD* (0.016,VII-L; 0.009,VI-R) and at VI-R in *tlc1-BD* (P = 0.015; but not at VII-L, P = 0.072). Cdc13p binding was similar in WT and *tlc1Δ* cells (P = 0.08, VII-L; 0.06, VI-R).

We used ChIP to determine if the stem-bulge region is also essential for Est1p binding to telomeres (Figure 3). Est1p-Myc association with the VII-L telomere was determined in synchronized *tlc1-SD* cells (Figure 3A). Est1p association was low, 4-fold over background, although the difference between it and the no tag strain had high significance only at the 45 min timepoint (Figure 3A). This low association was not due to reduced Est1p levels (Figure 3C, lane labeled SD).

Because the WT and *tlc1* mutant strains proceeded similarly through the cell cycle, the level of Est1p telomere binding in asynchronous cultures can be used to compare Est1p binding in different backgrounds. In asynchronous cells, Est1-Myc binding in WT cells was 11.6 fold (telomere VII-L) or 9.4 fold (telomere VI-R) (Figure 3B). The level of Est1-Myc binding in asynchronous cells expressing the telomerase defective *tlc1-SD* (3.2 fold, VII-L; 2.0 fold, VI-R; lanes labeled SD) or *tlc1-BD* (3.3 fold, VII-L; 2.4 fold, VI-R; lanes labeled BD) alleles was not significantly different from binding in *tlc1Δ* cells (2.5 fold, VII-L; 3.0 fold, VI-R) (Figure 3B). In all three *tlc1* mutants, Est1p binding was low but significantly higher than in the no-tag control. In contrast, the telomerase proficient *tlc1-SC* allele supported high levels of Est1-Myc telomere binding, (9.9 fold, VII-L; 6.3 fold, VI-R; lanes labeled SC), a level that was not significantly different from WT. Low Est1-Myc binding was not due to difficulties detecting proteins at short telomeres as Cdc13p-Myc telomere binding was high in all backgrounds (Figure 3D). Est1p abundance was similar in WT and mutant strains (Figure 3C). We conclude that Est1p interaction with the conserved stem-bulge region of TLC1 RNA is required for normal levels of Est1p telomere binding.

### Est2p Telomere Binding in Late S Phase Requires the Stem-Bulge Region of TLC1 RNA

By co-immunoprecipitation, Est2p interacts normally with both TLC1-SD and TLC1-BD RNAs [24]. Although the late S/G2 peak of Est2-G8-Myc binding did not occur in *est1Δ* cells (Figure 1A), this effect could be due to an off-telomere effect of Est1p on Est2p structure or function that can not occur in the absence of Est1p. Alternatively, Est1p telomere binding may be required for Est2p binding. Since Est1p was present at normal levels (Figure 3C) but had low telomere association in *tlc1-SD* and *tlc1-BD* cells (Figure 3A, B), it is possible to distinguish between these possibilities in these strains.

**Figure 4:**
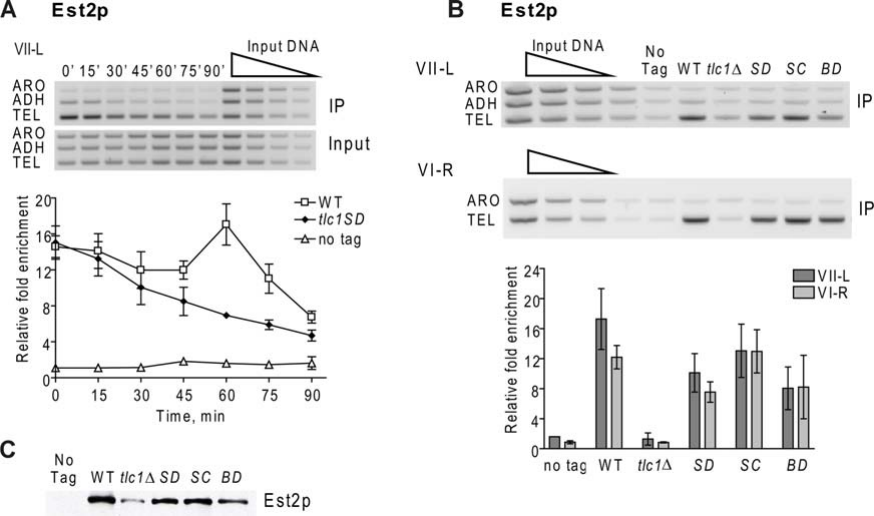
Est2p telomere binding in late S/G2 phase is reduced in mutants that lack the stem-bulge region of telomerase RNA. Methods are the same as in Figure 1 legend. A. Est2p binding to VII-L telomere in synchronous *tlc1-SD* (black diamonds) versus WT (white squares), or untagged (white triangles) cells. Est2p binding to the VII-L telomere was significantly lower in *tlc1-SD* than in WT cells late in the cell cycle (from 45 to 90 min, P values ranged from P = 0.0007 at 60 min to 0.018 at 90 min). B. Est2p binding to VII-L (top) or VI-R (bottom) telomeres in asynchronous WT and mutant cells. Bar graphs show average Est2p association with telomere VII-L (dark grey) or VI-R (light grey) with error bars indicating ±one standard deviation from that average; abbreviations for strains are SD, *tlc1-SD*; SC, *tlc1-SC*; and BD, *tlc1-BD*. The level of Est2p telomere binding in *tlc1-SD* cells was significantly lower than in WT (P = 0.004, VII-L; 0.0003, VI-R) as well as at the VII-L telomere in *tlc1-BD* cells (P = 0.001). The level of Est2p telomere binding in WT and *tlc1-SC* cells was not significantly different (P = 0.083, VII-L; 0.573, VI-R). C. Western analysis of Est2p-G8-Myc in extracts from WT and mutant strains. Abbreviations for strains are same as in panel B.

In synchronous *tlc1-SD* cells, Est2-G8-Myc binding to the VII-L telomere was very similar to the pattern of Est2-G8-Myc binding in *est1Δ* and *cdc13-2* cells (compare Figure 4A, *tlc1-SD*, black diamonds, to Figure 1A, *est1Δ*, black circles and 1B, *cdc13-2*, black triangles). That is, Est2-G8-Myc binding was at WT levels in G1 and early S phase but was significantly reduced in late S/G2 phase. Est2-G8-Myc binding was also determined in asynchronous cells expressing the TLC1 alleles (Figure 4B). As shown previously [19], Est2p binding was absolutely dependent on *TLC1*: Est2-G8-Myc binding was at background levels in *tlc1Δ* cells (enrichment of 1.3 fold, VII-L; 0.8 fold, VI-R). The high Est2-G8-Myc binding in WT cells (17.3 fold, VII-L, 12.2 fold VI-R) was significantly reduced but still detectable in asynchronous *tlc1-SD* cells (VII-L 10.1 fold; VI-R, 7.6 fold), consistent with normal Est2-G8-Myc binding throughout most of the cell cycle and reduced binding in late S/G2 phase. Similar results were seen in asynchronous *tlc1-BD* cells (enrichment at VII-L, 8.0 fold; VI-R, 8.2 fold). The reduction in Est2-G8-Myc telomere binding in *tlc1-SD* and *tlc1-BD* cells was not due to a reproducible decrease in Est2p abundance (Figure 4C). These results demonstrate that Est1p is needed in *cis* (i.e., at the telomere) to support WT levels of Est2p telomere binding in late S/G2 phase.

### Neither Est2p nor Est1p Is Telomere-Associated when Both the TLC1-Ku80p G1 and the TLC1-Est1p Late S/G2 Recruitment Pathways Are Mutated

**Figure 5:**
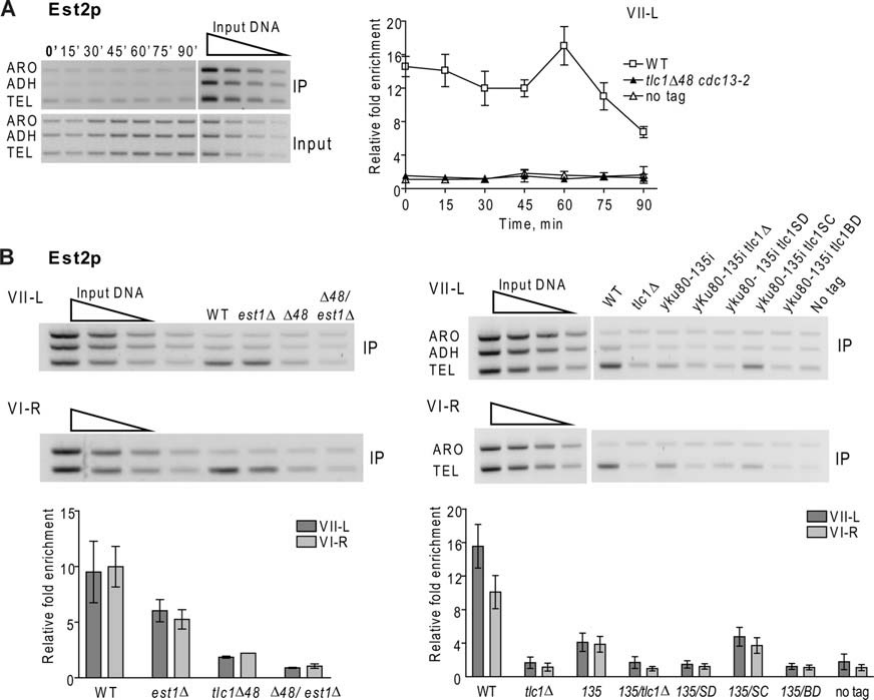
Est2p telomere binding is eliminated in mutants lacking both the G1 and the late S/G2 phase recruitment pathways. Methods are the same as in Figure 1 legend. A. Est2p binding to VII-L telomere in synchronous *tlc1Δ48 cdc13-2* (black triangles) versus WT (white squares), or untagged (white triangles) cells. Est2p telomere binding in the double mutant was not different from the no-tag control (P = 0.11 to 0.85) except at the 0 min time point (P = 0.039). B. Est2p binding to VII-L (top) or VI-R (bottom) telomeres in asynchronous WT and mutant cells. Bar graphs show average Est2p association with telomere VII-L (dark grey) or VI-R (light grey). Abbreviations are *Δ48*, *tlc1Δ48*; *13-2*, *cdc13-2*; *135*, *yku80-135i*, SD, *tlc1-SD*; SC, *tlc1-SC*; BD, *tlc1-BD*. For double mutants, there is a slash between the two alleles as in *Δ48/est1Δ* which stands for *tlc1Δ48 est1Δ*. Error bars indicating ±one standard deviation from that average. Est2p binding was indistinguishable in *tlc1Δ* versus the double mutants *yku80-135i tlc1-SD* or *yku80-135i tlc1-BD* cells (P values ranged from 0.28 to 0.85) while Est2p binding in the double mutant *yku80-135i tlc1-SC* was significantly greater than in *tlc1Δ* (P = 0.003, VII-L; 0.0001, VI-R).

**Figure 6:**
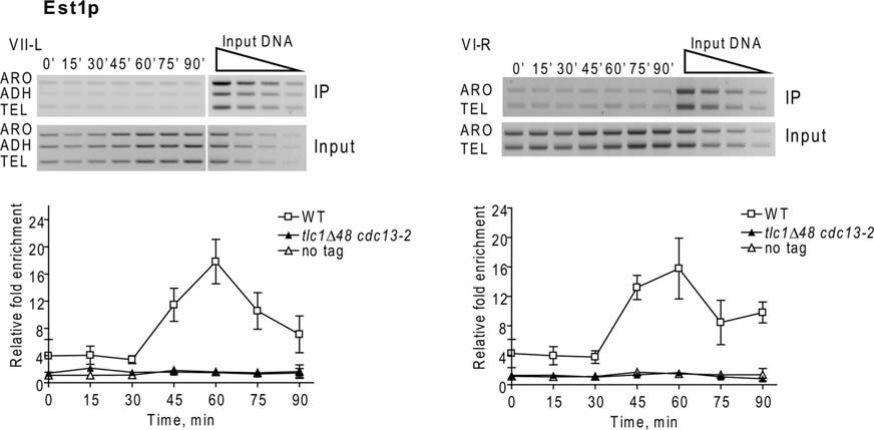
Est1p telomere binding is eliminated in mutants lacking both the G1 and the late S/G2 phase recruitment pathways. Methods are the same as in Figure 1 legend. Est1p binding to VII-L (left) or VI-R (right) telomeres in synchronous *tlc1Δ48 cdc13-2* (black triangles) versus WT (white squares), or untagged (white triangles) cells. Est1p telomere binding in the double mutant *tlc1Δ48 cdc13-2* was indistinguishable from the no-tag control at all time points at the VII-L telomere (P ranged from 0.12 to 0.98) except at 15 min (P = 0.0007). Est1p binding in the double mutant was indistinguishable at the VI-R telomere (P values ranged from 0.38 to 0.64) except at 15 (P = 0.0423) and 30 (P = 0.0399) mins.

Eliminating the specific interaction between Yku80p and TLC1 RNA with the *tlc1Δ48* or *yku80-135i* mutations [21],[23] eliminates Est2p at the telomere in G1 and early S phase [21]. Mutations that strongly reduced the amount of telomere bound Est1p, such as *cdc13-2*, *tlc1-SD* and *tlc1-BD*, or an *est1Δ* allele that eliminates Est1p altogether, lack high levels of Est2p binding in late S/G2 phase [19]; (Figure 1A, B; 4A). To determine if these pathways are the only ones that recruit telomerase to yeast telomeres, Est2p telomere binding was examined in double mutants that eliminate both pathways. Est2-G8-Myc binding was determined in synchronous *tlc1Δ48 cdc13-2* cells (Figure 5A). Although Est2p abundance was normal in this background (Figure 1C, lane DM, double mutant), Est2-G8-Myc binding to the VII-L telomere (Figure 5A, black triangles) was not significantly different from the no tag control (Figure 5A, white triangles). Est2-G8-Myc binding was also very low at both the VII-L and VI-R telomeres in asynchronous *yku80-135i tlc1-SD*, *yku80-135i tlc1-BD* and *tlc1Δ48 est1Δ* cells (Figure 5B). Est1-Myc binding to both the VII-L and VI-R telomeres was indistinguishable from the no-tag control strain in synchronous *tlc1Δ48 cdc13-2* cells (Figure 6). We conclude that the TLC1-Ku mediated pathway that recruits Est2p to telomeres in G1 phase [21] and the pathway that requires specific interactions of Est1p with both Cdc13p (Figure 2A) and TLC1 RNA (Figure 3A, B) that maintains high levels of telomere bound Est2p in late S/G2 phase are the only pathways that recruit Est2p to DNA ends in otherwise WT cells.

## Discussion

A specific interaction between a 48 bp stem-loop region in TLC1 RNA and Yku80p brings Est2p to the telomere in G1 and early S phase [21]. This TLC1-Ku interaction is also required for WT levels of telomere-associated telomerase in late S/G2 phase as both Est2p (∼40–50% of WT) and Est1p (∼33% of WT) telomere binding are reduced in these backgrounds. Cells that lack the Ku-TLC1 RNA interaction (*tlc1Δ48*, *yku80-135i*, and *yku*Δ) have short but stable telomeres and do not senesce.

In contrast, four *est* strains examined here (*est1Δ*, *cdc13-2*, *tlc1-SD*, and *tlc1-BD*) as well as *est3Δ* cells (data to be published elsewhere), had WT levels of telomere associated Est2p in G1 and early S phase but reduced (∼50% of WT) Est2p telomere binding in late S/G2 phase (Figure 1A, B; 4A). The only *est* strain (other than *est2Δ*) that did not have this pattern was *tlc1Δ* in which there was no Est2p telomere binding at all [19] (Figure 4B). Reduced Est2p telomere binding was not associated with a marked reduction in Est2p abundance except in the *tlc1Δ* strain [19] (Figure 1C, 4C).

Est2p binding in late S/G2 phase was Est1p dependent (Figure 1A). However, the presence of Est1p was not sufficient for WT Est2p telomere association as Est2p binding was equally reduced in late S/G2 phase in *tlc1-SD* and *tlc1-BD* cells (Figure 4A, B) where Est1p was present (Figure 3C), but is neither TLC1-associated [24] nor telomere-bound (Figure 3A, B). The fact that Est2p does not bind telomeres at all in a *tlc1Δ* strain [19] (Figure 4B) is consistent with TLC1 driving Est2p telomere association throughout the cell cycle via specific interactions with other proteins, Ku80p in G1/early S phase and Est1p in late S/G2 phase.

The G-tail binding Cdc13p is not a telomerase subunit [28]. Therefore, the late S/G2 phase reduction in Est2p telomere binding in *cdc13-2* cells [19] (Figure 1B) is unlikely due to a change in telomerase structure. This strain also had low Est1p binding, ∼25% of WT levels (Figure 2A) yet Est1p and Est2p abundance was normal in *cdc13-2* cells (Figure 1C, 2B). These data can be explained if the holoenzyme comes to the telomere (or is held at the telomere) in late S/G2 phase via a specific interaction between Est1p and Cdc13p that is impaired in *cdc13-2* cells [17]. Low but detectable Est1p binding in *cdc13-2* cells is consistent with the effects of this mutation on telomerase recruitment to a double strand break (DSB) that is generated next to a tract of telomeric DNA. In *cdc13-2* cells, Est1p binding to the break was much lower than in WT cells for up to 2 hrs after DSB formation, but at 3 hrs, Est1p binding was >50% of the WT level [29]. Est1p telomere binding was also reduced when it was unable to interact with TLC1 RNA as in *tlc1-SD* and *tlc1-BD* cells (Figure 3A, B). The Cdc13p and TLC1 pathways for Est1p recruitment are not redundant as Est1p binding was low when either interaction was disrupted. However, the pathways must be somewhat independent as Est1p levels at the telomere in late S/G2 phase were ∼25% of WT in both *cdc13-2* (Figure 2A) and *tlc1-SD* cells (Figure 3A), higher than the background level of Est1p telomere binding in *est2Δ* (Figure 2C) and *tlc1Δ48 cdc13-2* cells (Figure 6). Likewise, in asynchronous cells, Est1p telomere binding was statistically indistinguishable in *tlc1Δ*, *tlc1-SD*, and *tlc1-BD* cells. However, in each strain, binding was higher in the mutant than in the no-tag control (Figure 3B). Thus, a small but significant amount of Est1p can associate with telomeres in the complete absence of TLC1 RNA. We speculate that this low level association is due to the Est1p-Cdc13p interaction.

Together with earlier findings, the data presented here support several conclusions. First, the G1 and the late S/G2 phase Est2p recruitment pathways must be the only ones that bring telomerase to yeast telomeres in otherwise WT cells since there was no telomere associated Est2p or Est1p in doubly mutant strains (*tlc1Δ48 cdc13-2*, *tlc1Δ48 est1Δ*, *yku80-135i tlc1-SD*, *yku80-135i tlc1-BD*; Figure 5, 6). Second, G1 bound Est2p was neither sufficient (e.g., *est1Δ*) nor necessary (e.g., *tlc1Δ48*) to maintain telomeres by telomerase. In fact, it is possible that the G1 recruitment pathway contributes to telomere length solely by protecting ends from degradation [30]. This view is supported by the finding that ≥60% of the Est2p that is telomere associated in G1 phase is located at least ∼100 bps from the chromosome end and thus is not in a position to lengthen telomeres [31]. Third, Est1p interacts poorly' with Cdc13p unless it is part of the holoenzyme. Three *est* mutants, *tlc1-SD*, *tlc1-BD* (Figure 3C) and *est2Δ* (Figure 2B) had WT levels of Est1p, but there was low (*tlc1-SD*, *tlc1-BD*; Figure 3A, B) or no (*est2Δ*; Figure 2C) Est1p at the telomere in these strains. Thus, *in vivo*, the interaction between Cdc13p and Est1p detected by biochemical and genetic methods [15],[16] either does not occur or is not stable at telomeres unless Est1p is part of the holoenzyme. Fourth, while reduced Est2p binding in late S/G2 phase correlated with an inability to maintain telomeres by telomerase, it was not sufficient to confer an *est* phenotype. The levels of telomere associated Est2p and Est1p in the non-senescing *tlc1Δ48*, *yku80-135i*, *ykuΔ* and *tel1Δ* strains [31],[32] are similar to what was seen here for four *est* mutants (Figure 1A, B; Figure 4A, B), yet telomeres in *tlc1Δ48*, *yku80-135i*, *ykuΔ*, and *tel1Δ* cells, while shorter than WT, are stable [22],[23],[33].

There are several mutually non-exclusive explanations for why similarly low levels of telomerase in late S/G2 phase support telomerase proficiency in some backgrounds (eg., *tel1Δ*, *tlc1Δ48*, *yku80-135i*, *ykuΔ*) and an *est* phenotype in others (*cdc13-2*, *tlc1-BD*, *tlc1-SD*). For example, there may be fairly subtle quantitative differences between Est2p and/or Est1p binding between the two mutant classes that are not detected by ChIP. Alternatively, there may be qualitative differences between the telomerase that is telomere associated in the two classes of mutants, such as post translational modification of telomerase subunits or the presence of Est3p. Another possibility is that telomere structure is different between *EST* and *est* strains in late S/G2 phase, and the telomeric structure found in *est* cells makes it harder for low amounts of telomerase to engage properly with chromosome ends. The idea that a specific telomere structure is required for telomerase activity is supported by the observation that forced association of Est1p and Est3p with Est2p in G1 phase cells is not sufficient to support telomerase-mediate telomere elongation [18].

From yeasts to humans, the amount of telomerase per cell is surprisingly low [34],[35]. Reducing this already low level by mutation in one of several telomerase components [36]–[39] or by altering a telomere structural protein [40] can cause fatal diseases, such as dyskeratosis congenita or idiopathic pulmonary fibrosis [41],[42]. Here we show that in some genetic backgrounds ∼50% of WT levels of Est2p at yeast telomeres in late S/G2 phase is not sufficient to maintain telomeric DNA and prevent cellular senescence. An understanding of why yeast cells are sensitive to reduced levels of telomerase in some genetic backgrounds but not in others may help clarify why even relatively modest reductions in telomerase levels in human stem cells affects their survival.

## Materials and Methods

### Yeast Strains and Plasmids

All experiments were carried out in YPH499 [43] background that was modified by insertion of *URA3* immediately adjacent to the left telomere of chromosome VII [44] to generate YPH499-UT, and the *BAR1* gene was deleted and replaced with *kanMX6* [21]. Proteins were epitope tagged at their endogenous loci as described [11],[21],[25] in a manner that places *TRP1* at the tagged locus. Briefly, Est1 was tagged at its carboxyl terminus with nine Myc epitopes [19], and Est2p was tagged at its carboxyl end with a Gly8 linker followed by 18 Myc epitopes [21],[25]. Cdc13p was tagged at its carboxyl terminus with 9 Myc epitopes [19]. Complete deletions of *TLC1* (replaced by *LEU2*), *EST1* (replaced by *HIS3*), and *EST2* (replaced by *HIS3*) were generated using PCR-mediated transformation [45]. The *TLC1* alleles *tlc1-SD* (stem disruption; 3 bp disrupted in predicted stem), *tlc1-SC* (stem disruption compensatory; potential for base-pairing restored to *tlc1-SD*), and *tlc1-BD* (bulge deletion; deletion of 5 bulged nucleotides) described in [24] were generously provided by Tom Cech and introduced by integration into the genome. The *tlc1Δ48* and *yku80-135i* alleles are described in [23] and were generously supplied by Dan Gottschling. The *cdc13-2* [16], *est1Δ*, *tlc1Δ*, *est2Δ*, *tlc1Δ48*, and *yku80-135i* mutations were generated as heterozygous diploids expressing Myc-tagged proteins. Doubly mutant strains were derived from heterozygous diploids at both loci that also expressed the desired Myc-tagged protein. In both cases, the heterozygous diploids were sporulated. Freshly dissected spores of the desired genotypes were identified by replica plating, grown up, and used immediately for ChIP analyses.

### Synchrony and ChIP Methods

The ChIP experiments were carried out, analyzed by multiplex PCR, and quantified exactly as described [21]. Briefly, relative fold enrichment of a protein with telomeres was determined by (TEL_IP_/ARO_IP_)/(TEL_input_/ARO_input_) where input is the amount of the DNA sequence that was PCR amplified in the samples before precipitation and IP is the amount of the sequence in the anti-Myc immuno-precipitate. Cell synchrony experiments were carried out as in [21]. Briefly, 30°C grown, log phase cells (A660 = 0.3) cells were arrested in late G1 phase using alpha factor (Sigma), removed from alpha factor (0 minutes), and then allowed to proceed through a synchronous cell cycle at 24°C. Samples were removed at 15 min intervals and processed for ChIP and FACS (fluorescent activated cell sorting) analysis at each time point. For synchrony experiments, the data for each time point are presented as the mean of the three or more independent synchrony experiments plus or minus one standard deviation from the mean. Likewise, values for asynchronous cultures are the mean plus or minus one standard deviation for three independent cultures. Statistical significance was determined using a two-tailed Student's t test. For the purposes of this paper, P values ≤0.05 were considered significant.

## References

[1] Vega L, Mateyak M, Zakian V (2003). Getting to the end: telomerase access in yeast and humans.. Nat Rev Mol Cell Biol.

[2] Lundblad V, Szostak JW (1989). A mutant with a defect in telomere elongation leads to senescence in yeast.. Cell.

[3] Wellinger RJ, Wolf AJ, Zakian VA (1993). Origin activation and formation of single-strand TG_1–3_ tails occur sequentially in late S phase on a yeast linear plasmid.. Mol Cell Biol.

[4] Wellinger RJ, Wolf AJ, Zakian VA (1993). *Saccharomyces* telomeres acquire single-strand TG_1–3_ tails late in S phase.. Cell.

[5] Wellinger RJ, Ethier K, Labrecque P, Zakian VA (1996). Evidence for a new step in telomere maintenance.. Cell.

[6] Diede SJ, Gottschling DE (1999). Telomerase-mediated telomere addition *in vivo* requires DNA primase and DNA polymerases alpha and delta.. Cell.

[7] Marcand S, Brevet V, Mann C, Gilson E (2000). Cell cycle restriction of telomere elongation.. Curr Biol.

[8] Lin JJ, Zakian VA (1996). The *Saccharomyces CDC13* protein is a single-strand TG_1–3_ telomeric DNA binding protein *in vitro* that affects telomere behavior *in vivo*.. Proc Natl Acad Sci USA.

[9] Nugent CI, Hughes TR, Lue NF, Lundblad V (1996). Cdc13p: a single-strand telomeric DNA-binding protein with a dual role in yeast telomere maintenance.. Science.

[10] Bourns BD, Alexander MK, Smith AM, Zakian VA (1998). Sir proteins, Rif proteins and Cdc13p bind *Saccharomyces* telomeres *in vivo*.. Mol Cell Biol.

[11] Tsukamoto Y, Taggart AKP, Zakian VA (2001). The role of the Mre11-Rad50-Xrs2 complex in telomerase-mediated lengthening of *Saccharomyces cerevisiae* telomeres.. Curr Biol.

[12] Garvik B, Carson M, Hartwell L (1995). Single-stranded DNA arising at telomeres in cdc13 mutants may constitute a specific signal for the *RAD9* checkpoint.. Mol Cell Biol.

[13] Grandin N, Damon C, Charbonneau M (2001). Ten1 functions in telomere end protection and length regulation in association with Stn1 and Cdc13.. EMBO J.

[14] Grandin N, Reed SI, Charbonneau M (1997). Stn1, a new *Saccharomyces cerevisiae* protein, is implicated in telomere size regulation in association with Cdc13.. Genes Dev.

[15] Qi H, Zakian VA (2000). The *Saccharomyces* telomere-binding protein Cdc13p interacts with both the catalytic subunit of DNA polymerase α and the telomerase-associated Est1 protein.. Genes Dev.

[16] Pennock E, Buckley K, Lundblad V (2001). Cdc13 delivers separate complexes to the telomere for end protection and replication.. Cell.

[17] Evans SK, Lundblad V (1999). Est1 and Cdc13 as comediators of telomerase access.. Science.

[18] Osterhage JL, Talley JM, Friedman KL (2006). Proteasome-dependent degradation of Est1p regulates the cell cycle-restricted assembly of telomerase in *Saccharomyces cerevisiae*.. Nat Struct Mol Biol.

[19] Taggart AKP, Teng S-C, Zakian VA (2002). Est1p as a cell cycle-regulated activator of telomere-bound telomerase.. Science.

[20] Gallardo F, Olivier C, Dandjinou AT, Wellinger RJ, Chartrand P (2008). TLC1 RNA nucleo-cytoplasmic trafficking links telomerase biogenesis to its recruitment to telomeres.. EMBO J.

[21] Fisher T, Taggart A, Zakian V (2004). Cell cycle-dependent regulation of yeast telomerase by Ku.. Nat Struct Mol Biol.

[22] Peterson SE, Stellwagen AE, Diede SJ, Singer MS, Haimberger ZW (2001). The function of a stem-loop in telomerase RNA is linked to the DNA repair protein Ku.. Nat Genet.

[23] Stellwagen AE, Haimberger ZW, Veatch JR, Gottschling DE (2003). Ku interacts with telomerase RNA to promote telomere addition at native and broken chromosome ends.. Genes Dev.

[24] Seto AG, Livengood AJ, Tzfati Y, Blackburn EH, Cech TR (2002). A bulged stem tethers Est1p to telomerase RNA in budding yeast.. Genes Dev.

[25] Sabourin M, Tuzon C, Fisher T, Zakian V (2007). A flexible protein linker improves the function of epitope-tagged proteins in *Saccharomyces cerevisiae*.. Yeast.

[26] Lin J-J, Zakian VA (1995). An *in vitro* assay for *Saccharomyces* telomerase requires *EST1*.. Cell.

[27] Steiner BR, Hidaka K, Futcher B (1996). Association of the Est1 protein with telomerase activity in yeast.. Proc Natl Acad Sci USA.

[28] Hughes TR, Evans SK, Weilbaecher RG, Lundblad V (2000). The Est3 protein is a subunit of yeast telomerase.. Curr Biol.

[29] Bianchi A, Negrini S, Shore D (2004). Delivery of yeast telomerase to a DNA break depends on the recruitment functions of Cdc13 and Est1.. Mol Cell.

[30] Vega L, Phillips J, BR T, DP T, Onigbanjo M (2007). Sensitivity of yeast strains with long G-tails to levels of telomere-bound telomerase.. PLoS Genet.

[31] Sabourin M, Tuzon C, VA Z (2007). Telomerase and Tel1p preferentially associate with short telomeres in *S. cerevisiae*.. Mol Cell.

[32] Goudsouzian L, Tuzon C, Zakian VA (2006). *S. cerevisiae* Tel1p and Mre11p are required for normal levels of Est1p and Est2p telomere association.. Mol Cell.

[33] Greenwell PW, Kronmal SL, Porter SE, Gassenhuber J, Obermaier B (1995). TEL1, a gene involved in controlling telomere length in *S. cerevisiae*, is homologous to the human ataxia telangiectasia gene.. Cell.

[34] Cohen SB, Graham ME, Lovrecz GO, Bache N, Robinson PJ (2007). Protein composition of catalytically active human telomerase from immortal cells.. Science.

[35] Mozdy AD, Cech TR (2006). Low abundance of telomerase in yeast: implications for telomerase haploinsufficiency.. RNA.

[36] Mitchell JR, Wood E, Collins K (1999). A telomerase component is defective in the human disease dyskeratosis congenita.. Nature.

[37] Vulliamy T, Beswick R, Kirwan M, Marrone A, Digweed M (2008). Mutations in the telomerase component NHP2 cause the premature ageing syndrome dyskeratosis congenita.. Proc Natl Acad Sci USA.

[38] Vulliamy T, Marrone A, Goldman F, Dearlove A, Bessler M (2001). The RNA component of telomerase is mutated in autosomal dominant dyskeratosis congenita.. Nature.

[39] Marrone A, Walne A, Tamary H, Masunari Y, Kirwan M (2007). Telomerase reverse-transcriptase homozygous mutations in autosomal recessive dyskeratosis congenita and Hoyeraal-Hreidarsson syndrome.. Blood.

[40] Savage SA, Giri N, Baerlocher GM, Orr N, Lansdorp PM (2008). TINF2, a component of the shelterin telomere protection complex, is mutated in dyskeratosis congenita.. Am J Hum Genet.

[41] Armanios MY, Chen JJ, Cogan JD, Alder JK, Ingersoll RG (2007). Telomerase mutations in families with idiopathic pulmonary fibrosis.. N Engl J Med.

[42] Tsakiri KD, Cronkhite JT, Kuan PJ, Xing C, Raghu G (2007). Adult-onset pulmonary fibrosis caused by mutations in telomerase.. Proc Natl Acad Sci USA.

[43] Sikorski RS, Hieter P (1989). A system of shuttle vectors and yeast host strains designed for efficient manipulation of DNA in *Saccharomyces cerevisiae*.. Genetics.

[44] Gottschling DE, Aparicio OM, Billington BL, Zakian VA (1990). Position effect at *S. cerevisiae* telomeres: reversible repression of Pol II transcription.. Cell.

[45] Lorenz MC, Muir RS, Lim E, McElver J, Weber SC (1995). Gene disruption with PCR products in *Saccharomyces cerevisiae*.. Gene.

